# Recent Advances in Research on Iron Metabolism, Ferritin, and Hepcidin

**DOI:** 10.3390/ijms27020906

**Published:** 2026-01-16

**Authors:** Alessandro Polizzi

**Affiliations:** Unità Operativa di Patologia Clinica, Ospedali Riuniti di Vittoria e Comiso-A.S.P Ragusa, Via Papa Giovanni XXIII Vittoria, 97019 Ragusa, Italy; alessandro.polizzi@asp.rg.it

**Keywords:** iron, hepcidin, ferroportin, ferritin, cell signaling

## Abstract

This study aimed to provide a synthesis of current knowledge on iron homeostasis, focusing on major metabolic pathways and evolving research perspectives. A systematic review was conducted, analyzing the most relevant pathological conditions associated with iron metabolism, including iron overload and iron deficiency. Iron overload (IO) encompasses a wide range of disorders that lead to systemic iron accumulation and organ damage, while iron deficiency (ID) is characterized by insufficient iron availability for physiological needs. IO is dealt with a focused attention, exploring molecular mechanisms and emerging therapeutic strategies. In this context, hepcidin not only represents a valuable biomarker for iron overload but also serves as a potential target for novel therapies that are currently in the experimental phase. Conversely, for ID, both traditional biomarkers and recently proposed indicators help in diagnosing ID and correlating it with erythropoietic activity.

## 1. Introduction

Iron is involved in different cellular processes, from oxygen transport to DNA synthesis. Iron homeostasis is tightly controlled through a highly coordinated network of systemic and cellular mechanisms which regulate its absorption, transport, storage, and recycling. Ferritin, a ubiquitous intracellular protein, plays a fundamental role in cellular iron storage and protection against oxidative damage. It safely sequesters excess iron in a bioavailable form, thereby preventing toxicity while preserving iron for future metabolic demands. Dysregulation of ferritin expression is associated with inflammatory conditions, neurodegenerative diseases, and malignancies, emphasizing its importance beyond iron storage. Hepcidin is a hormone, that is produced by the liver and represents the main regulator of systemic iron homeostasis. It exerts its function by binding to ferroportin, allowing its internalization and degradation, thereby, effectively reducing iron export from enterocytes, macrophages, and hepatocytes. Hepcidin levels are tightly modulated by iron stores, inflammatory signals, erythropoietic activity, and hypoxia. Altered hepcidin expression is implicated in various iron-related disorders, including hemochromatosis, anemia of inflammation, and iron-loading anemias. Recent advances in molecular biology, structural biochemistry, and genomics have significantly expanded our understanding of the intricate regulatory networks governing iron metabolism. These developments have shed light on the dynamic interplay between ferritin, hepcidin, and ferroportin, and their pivotal roles in maintaining physiological iron balance. The aim of the paper is to investigate the role of ferritin and hepcidin in iron metabolism, analyzing their role in pathological conditions of iron overload and iron deficiency.

## 2. Iron Cycle: From Absorption to Body Distribution

Due to its ability to donate and accept electrons, iron can be found in two oxidation states: ferric or trivalent iron, and ferrous or divalent iron. The daily requirement of iron in humans is about 25–30 mg. Dietary iron could be divided into two forms: heme and non-heme bound iron. Heme iron is mainly contained in meat and poultry, while cereals and vegetables are sources of non-heme-bound iron.

**Figure 1:**
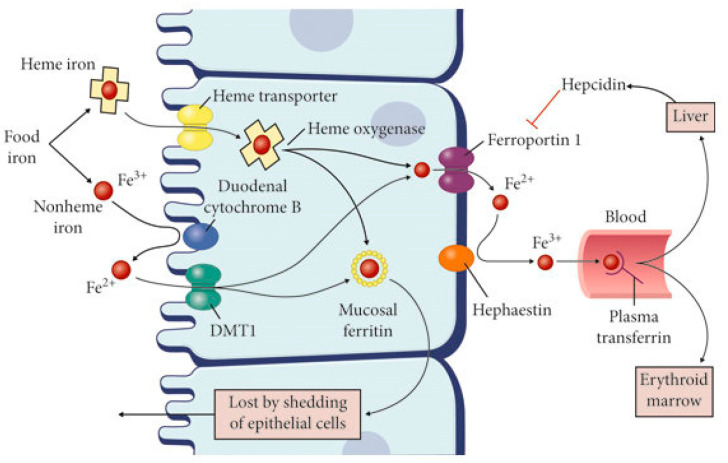
Intestinal iron absorption. Figure inserted from [3]. This process occurs mainly in the duodenum and the proximal jejunum. Ferric iron is reduced to ferrous iron thanks to cytochrome B. In this way, it can enter so that it can reach the luminal side of enterocyte through the DMT-1 pathway. Once into the enterocyte, ferrous iron is oxidized to ferric iron by hephaestin, so as to be transported by the transferrin into the bloodstream. Hepcidin from the liver inactivates ferroportin, thereby inhibiting iron uptake. Abbreviations: DMT1-divalent metal transporter 1; HCP1 – heme carrier protein 1; TfR1-transferrin receptor-1; DCYTB- duodenal cytochrome B reductase; FPN1-ferroportin.

The absorption phase mainly concerns divalent iron. It takes place in the apical membrane of enterocytes and can involve different cellular systems: from transferrin–transferrin receptor system to erythrophagocytosis, until the divalent metal transporter 1 (DMT 1) (Figure 1) [1].

Instead, the trivalent iron is not easily absorbed; initially, it should be reduced to the divalent one, thanks to duodenal cytochrome B (DCYTB) reductase [2]. Once absorbed, the divalent iron could be transported or stored, based on the requirements of the organism. Iron storage takes place in the hepatocytes, where iron is safekept in ferritin (FT). The export of iron could be achieved, firstly, through the iron oxidation to the trivalent ionic form, with the subsequent introduction into the bloodstream through the ferroportin (FPN) [3]; alternative pathways involve heme export and ferritin secretion [4].

FPN is the iron transporter expressed in mammalian cells on the basolateral membrane of duodenal enterocytes, on macrophages and hepatocytes, where it plays a crucial role in controlling iron release. It is composed of 12 transmembrane helices divided into two halves forming two lobes [5]. Once iron is oxidized to the trivalent ionic form, by oxygen-dependent ferroxidases, it is then transferred into transferrin (Tf), a glycoprotein involved in the transportation of iron into the bloodstream to various organs and tissues [2]; if ferroxidase is missing or deficient, the divalent iron is not exported, and it is stored within ferritin. Under normal conditions, most of the circulating iron is bound to transferrin. In some pathological conditions, when the iron binding capacity of transferrin (Tf) is exceeded, the non-transferring bound iron (NTBI) may be present. A component of NTBI is the labile plasma iron (LPI), which can be captured by non-hematopoietic cells, resulting in parenchymal iron accumulation and free radical damage to tissues [6]. The transferrin–trivalent iron complex circulating in the bloodstream enters the cells through the cell-surface transferrin receptor, also called transferrin receptor-1 or TfR1. Only iron-saturated transferrin, called diferric transferrin, is recognized by TfR and subsequently internalized through vesicles, called endosomes, where the trivalent iron is again reduced to the divalent ionic form through DMT 1. In this way, the divalent iron could be incorporated in storage proteins, while transferrin could be released back into the bloodstream [7].

## 3. Discussion

### 3.1. Ferritin

Ferritin is found in the liver, spleen and bone marrow. This protein provides the correct storage or distribution of iron when it is required. Ferritin is structurally composed of two chains [8]: the heavy chain (FTH) and light chain (FTL). FTH has ferroxidase activity, which transforms iron from the divalent to the trivalent ionic form, thanks to a binuclear iron-binding site; in this way, it could enter the ferroxidase site. FTL, instead, is focused on mineralization and iron storage. The main molecular regulation of ferritin takes place thanks to two proteins: the iron regulatory proteins (IRPs) and the iron responsive elements (IREs), in the so-called IRE-IRP system [9]. IRPs monitor the cellular iron levels; IREs encodes for proteins related to iron metabolism: it can bind to the 5′ untranslated region (5′ UTR) of ferritin, inhibiting the translation of the mRNA, or in the 3′ UTR, so that the translation of mRNA can be allowed. In case of iron depletion, IRPs bind to IREs in the 50 UTR, impairing the translation of the mRNA, allowing the availability of iron [10]. Ferritin lacks the classical secretion through the endoplasmic reticulum–Golgi system but presents an untraditional lysosomal secretion pathway.

### 3.2. Ferritinophagy

Iron cellular homeostasis could be regulated by two processes: ferritinophagy and ferroptosis.

**Figure 2:**
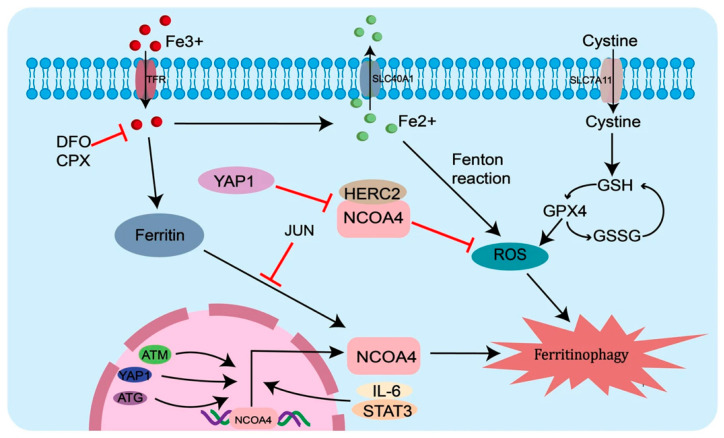
The ferritinophagy pathway. Inside the nucleus, ATM gene can promote interaction between NCOA4 and ferritin by phosphorylating NCOA4. Inside the cytoplasm, NCOA4 binds to FTH1 and drives it to lysosome, releasing ferrous iron, promoting ferritinophagy. The interacton between NCOA4 and FTH1 can be blocked by YAP1 protein, which can downregulate glutathione peroxidase 4 (GPX4), FTH1, and SLC7A11. Autophagy-associated genes (ATGs) can activate NCOA4, whose overexpression promotes intracellular iron ion transportation and cellular ferritinophagy. Furthermore, NCOA4 is also regulated by the IL-6/STAT3 signaling pathway, which protects from ferritinophagy inhibiting STAT3. When the JAK2/STAT3 pathway is activated, such as IL-6, JAK2 phosphorylates STAT3; when in the nucleus, this binds to the promoter region of SLC7A11, upregulating SLC7A11, neutralizing ROS, and inhibiting lipid peroxidation. Abbreviations: ATM—ataxia telangiectasia mutated gene; YAP1—Yes-associated protein 1; HERC2—E3 ubiquitin protein ligase antibody; GSH—Glutathione; GSSG—glutathione disulfide; 1L-6—Interleukin 6; STAT3—signal transducer and activator of transcription; ATG—genes autophagy related; SLC7A11—solute Carrier Family 7 (Cationic Amino Transported system) Member 11. Figure inserted from [18].

Ferritinophagy is based on a selective autophagy of ferritin, particularly in case of excess intracellular ferritin, and low intracellular iron levels, with the aim to increase iron levels. In case of iron depletion, IRPs bind to IREs; this promotes the binding of the corepressor of endoplasmic reticulum (RE) to the ferritin promoter region [11]. In this way, ferritin subunited on the C terminal of FTH becomes the binding site of an autophagy receptor, localized on the cargo nuclear receptor coactivator 4 (NCOA4) [12], forming an autophagosome which will bind the receptor LC3II in the lysosome, releasing iron into them [13]. Mutations implied in silencing or overexpression of NCOA4 can produce alterations in iron metabolism: in the case of NCOA4 upregulation, it could promote ferritinophagy, and in the case of NCOA4 downregulation, it may cause low iron bioavailability [14]. Sun et al. discovered that NCOA4 activity is regulated by the JNK-JUN pathway: JUN binds the promoter of NCOA4, inhibiting the interaction between NCOA4 and ferritin, while at the same time increasing ferritin autophagic degradation [15]. However, when iron is excessively released from ferritinophagy, after overexpression of NCOA4, a cytosolic pathway is created, readying the interaction between NCOA4 and HERC2 for ubiquitination and degradation of ferritin into lysosomes [16,17,18] (Figure 2). As a result, the accumulation of lipid peroxide, from the oxidation of polyunsaturated fatty acids, triggers an iron-dependent non-apoptotic cell death called ferroptosis [19].

### 3.3. Ferroptosis

Ferroptosis an iron-dependent cell death, different from apoptosis and necrosis, involving three kinds of metabolic pathways: thiol, lipid, and iron, along with lipid iron-dependent peroxidation until cell death. There are three hallmarks of ferroptosis: oxidation of polyunsaturated fatty acid-containing phospholipids (PUFA-PLs), accumulation of redox-active iron, and loss of lipid peroxide repair capacity [20].

**Figure 3:**
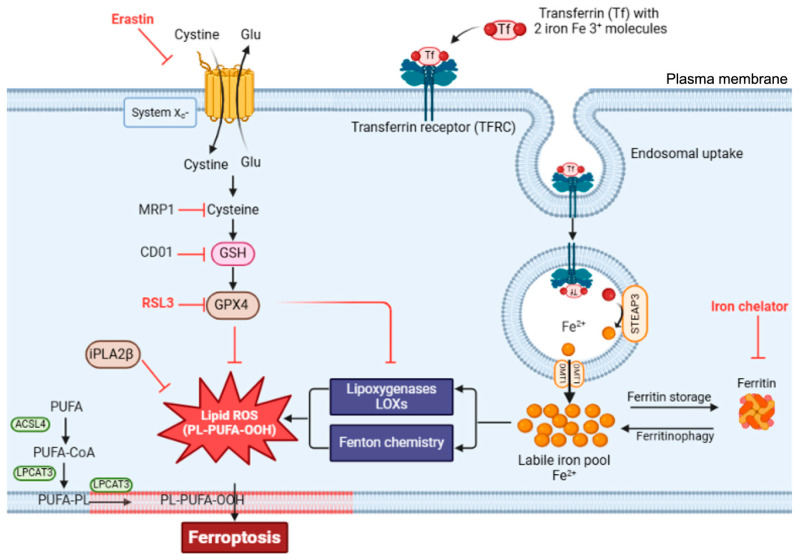
Mechanism underlying ferroptotic cell death. Ferroptosis begins with inhibition of the Xc−-GSH-GPX4 axis and the accumulation of intracellular free iron. Iron is imported into cells after binding to the transferrin receptor (TFR), forming the TFR complex (TFRC). Once inside the cell, ferric iron is converted to ferrous state and later imported into the cytoplasm. There, it can participate in redox reactions under the labile iron pool, or can be sequestered and stored in a redox-inactive form by ferritin. Iron chelators such as deferoxamine inhibit ferroptosis by reducing the labile iron pool and preventing iron-catalyzed oxidative damage. Erastin promotes ferroptosis by inhibiting system Xc^−^ or GPX4, resulting in lipid peroxide accumulation [21]. Abbreviations: DMT1- divalent metal transporter 1; FPN-ferroportin; LOX-lipoxygenase; ROS-reactive oxygen species; STEAP3-six-transmembrane epithelial antigen of the prostate 3; Tf-transferrin; TFRC-transferrin–receptor complex. Figure inserted from [20].

The antioxidant system which prevents ferroptosis involves the system called Xc−-GSH-GPX4 axis, which is composed of the cystine/glutamate antiporter system Xc−, glutathione (GSH), and glutathione peroxidase 4 (GPX4). The antiporter system reduces cystine to cysteine; this is an essential precursor for GSH synthesis, with GSH acting as the reducing substrate for GPX4. The latter is the main antioxidant enzyme, which converts toxic lipid hydroperoxides into nontoxic lipid, thus inhibiting the accumulation of toxic lipid ROS during ferroptosis (Figure 3).

Regarding the involvement of PUFA-PLs, long-chain fatty acyl-CoA synthetase 4 (ACSL4) and lysophosphatidylcholine acyltransferase 3 (LPCAT3) trigger the synthesis of arachidonic acid-phosphatidylethanolamines (PE-AA), whose oxidation leads to cell death. The presence of free iron or iron-containing lipoxygenase enzymes causes the oxidation of membrane PUFAs, resulting in the production of lipid ROS. The involvement of PUFA in ferroptosis has been proven by the accumulation of lipid peroxides, which could react with different molecules, from DNA to other proteins, leading to changes in membrane permeabilization and causing membrane instability. As the toxicity of iron-catalyzed PL-PUFA is induced by oxidation, ferroptosis could be prevented by small-molecule lipophilic antioxidants, such as vitamin E and coenzyme Q10, thanks to alternative endogenous antioxidant pathways, which catalyze the regeneration of ubiquinone, CoQ10, acting as a lipid peroxyl radical trap. There are also exogenous lipophilic antioxidants (e.g., ferrostatin-1) which could inhibit ferroptosis by preventing oxidative damage within the membrane, thereby reducing lipid hydroperoxides from PUFA-PLs. Ferroptosis could also be regulated by activated protein kinase (AMPK) via AMPK-mediated phosphorylation of acetyl-CoA carboxylase (ACC) and polyunsaturated fatty acid biosynthesis. The main regulator of this pathway is the liver kinase B1 (LKB1), which activates AMPK; in case of depletion of LKB1, it could activate lipid hydro peroxidation and lead to ferroptosis [21].

Although ferroptosis and ferritinophagy are distinct processes, they are both involved in the regulation of iron metabolism in cells. Ferroptosis can lead to the release of iron from cells, while ferritinophagy can increase the availability of iron for cellular use. The precise relationship between these two processes and their role in iron metabolism is an active area of research [22].

### 3.4. Hepcidin-/Ferroportin Axis

**Figure 4:**
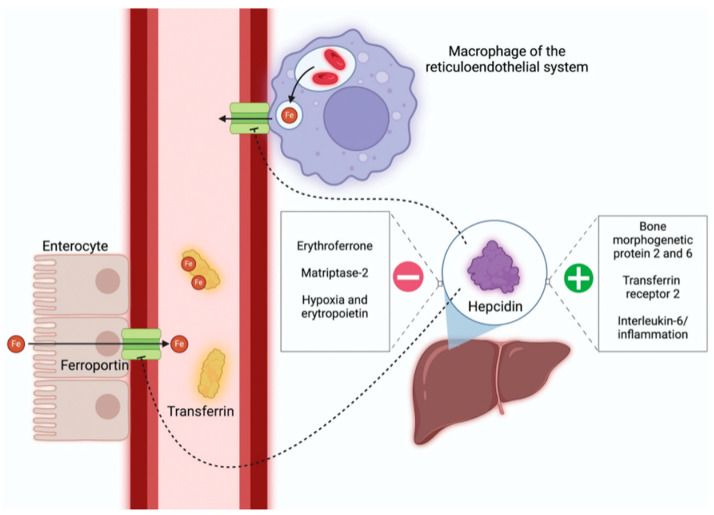
Representation of hepcidin-/ferroportin axis. After iron absorption into the enterocytes, plasma is accessed through the iron-exporter ferroportin. Macrophages of the reticuloendothelial system (RES) lyse senescent erythrocytes, recycling iron from catabolized heme. Hepcidin promotes the degradation of ferroportin, resulting in decreased plasma iron levels and iron retention in the RES. Figure inserted with from [26].

Ferroportin (FPN) is a protein responsible for iron transportand is expressed in different cellular elements. It comprises 12 transmembrane helices [5], which can change between two conformational states. In steady state conditions, FPN controls iron release from enterocytes, liver hepatocytes and macrophages. FPN expression at the post-translational level is regulated by circulating hepcidin. The latter is a 25-amino acid hormone, mainly produced in the hepatocytes, which limits the release of iron in the bloodstream, also promoting the reduction of iron export acting on ferroportin. To this aim, hepcidin reduces iron absorption in the duodenum [7] by downregulating iron absorption in the duodenum, blocking the release of iron from macrophages into the circulation, and controlling the transport of iron stores into hepatocytes. When the blood iron concentration increases, hepcidin mRNA transcription in liver tissues increases, while, in case of iron deficiency, hepcidin increases the iron absorption, leading to the release of iron from stores. Hepcidin expression involves several proteins present on the plasma membrane of hepatocytes, which constitute the iron-sensing complex: human hemochromatosis protein (HFE), transferrin receptor 2 (TfR2), hemojuvelin (HJV) and bone morphogenetic protein-6 (BMP-6) [23]. These proteins are affected by the amount of iron stored in the liver and the circulating iron, as iron-bound transferrin (holotransferrin). Holotransferrin binds to its receptor on the hepatocyte, where it can make a molecular complex with HJV. In this stage, intracellular signaling via the Small Mothers of Decapentaplegic (SMAD) phosphorylation pathway begins, with increased expression of *hepcidin* genes. In the case of iron deficit, increased TFR1 binds to HFE and inhibits the activation of the HFE-TFR2 complex, which prevents the production of hepcidin from being induced. TFR2 is like TFR1, except that it is produced by hepatocytes and is only found in the liver [24]. Moreover, it was discovered that BMP6 is a positive regulator of the hepcidin expression and the lack of BMP6 led to an excess of iron in the body [25,26]. In contrast, an overproduction of hepcidin is associated with iron-restricted anemia. Hepcidin production is also regulated by the erythropoiesis, as well as during pregnancy due to the increased iron requirements (Figure 4). Recently, the link between the hormone erythroferrone (ERFE) and iron balance has been discovered. ERFE is synthesized by erythroblasts in the bone marrow and spleen as a response to erythropoietin (EPO) stimulation [27]; it regulates the BMP-SMAD signal pathway by inhibiting its expression. High levels of erythroferrone were found in the pathogenesis of iron-loading anemias, making it a promising therapeutic target. Molecules that stimulate hepcidin production by inhibiting erythroferrone have demonstrated efficacy in restoring iron homeostasis. Furthermore, ERFE suppresses the transcription of hepcidin in hepatocytes, resulting in increased iron availability in conditions characterized by greater iron request [28], such as pregnancy [29] and in patients with beta-thalassemia and iron-deficiency anemia [30]. In a study conducted by Semercioglu et al., hepcidin levels were found to be significantly lower in children with iron-deficiency anemia than in children without it [31].

### 3.5. Iron Deficiency

ID is commonly found worldwide and is characterized by insufficient iron availability compared to the body’s needs and can be present with or without anemia [32]. Conventionally, the most prone to ID are people affected by malnutrition, conditions characterized by high iron demands such as pregnancy, or in cases of chronic blood loss. Furthermore, special attention is being paid to the patients affected by inflammatory conditions because they may have decreased iron stores, such as chronic heart failure (CHF), chronic kidney disease (CKD), and inflammatory bowel disease (IBD). The percentages of ID in these groups are different between the studies, because of the different definitions and patient selection criteria. Overall, however, about 50% of patients with CHF, 24–85% of patients with CKD, and 45% of patients with IBD are iron-deficient [33]. Finally, it must be emphasized that low serum iron, irrespective of ferritin and hemoglobin levels, has been associated with all-cause mortality as well.

### 3.6. Iron Overload

IO disorders represent various conditions that lead to systemic iron overload and organ damage. Hereditary hemochromatosis (HH) is an example of primary IO, caused by the mutation of the HFE gene, leading to hepcidin deficiency. Secondary IO may be associated with both hepcidin deficiency and hepcidin excess, leading to liver disease, iatrogenic iron administration, or hematologic conditions leading to ineffective erythropoiesis. The effects of these mutations can be divided into loss of function or gain of function. In the first case, iron export is impaired, resulting in iron accumulation in Kupffer cells. The second case leads to hepcidin resistance and the release of iron, as take place in hemochromatosis. Overall, these changes cause alterations in iron homeostasis, increasing iron absorption and high transferrin saturation. Conversely, in the pathology’s recessive form, hepcidin is excessively low and the severity of iron overload is related to the hormone deficiency [34]. In IO conditions, ferritin represents a valuable biomarker of total body iron. HH is characterized by mutations of the *HFE* gene, involved in the hepcidin/ferroportin axis, which results in insufficient hepcidin production or, exceptionally, in hepcidin resistance. Typical pathological signs include liver disease and pancreatic β-cell failure. The clinical severity of hemochromatosis is related to the increased toxicity from NTBI, which are toxic iron species bound to low molecular weight molecules. As a result, they cause the generation of reactive oxygen species (ROSs), resulting in cellular damage, liver fibrosis, chronic heart failure, diabetes, hypopituitarism, and other complications. Elevated transferrin saturation, >50% in men and >45% in women, is the first biochemical manifestation of the disease, which results from an inappropriately low level of circulating hepcidin.

### 3.7. Other Iron Loading-Related Conditions

Iron overload, as mentioned above, leads to the formation of ROS, resulting in insulin resistance through the downregulation of insulin receptor expression and worsening of glucose and lipid metabolism disorders. Moreover, an excess of fatty acids and lipotoxicity predisposes to inflammation and type 2 diabetes mellitus, inducing macrophage iron accumulation via FTH1 gene activation, which encodes the ferritin H subunit [35,36]. Unlike in patients with haemochromatosis, whose macrophages are typically iron depleted because of hepcidin deficiency, iron accumulation in macrophages has been associated with a pro-inflammatory condition [37], characterized by increased ferritin. This condition is defined as dysmetabolic iron overload syndrome (DIOS). DIOS is characterized by hyperferritinaemia, metabolic syndrome, insulin resistance, high serum ferritin (SF), and high transferrin saturation, but normal serum iron levels. Unlike patients with haemochromatosis, patients with DIOS exhibit reasonably preserved hepcidin production, but the presence of inherited variants, associated with impaired hepcidin, would favor the development of more severe iron accumulation. DIOS can be found in up to one third of patients with nonalcoholic fatty liver disease (NAFLD). A similar condition is the finding of hyperferritinaemia related to metabolic dysfunction (MHF), characterized by a variable degree of iron stores in the body, ranging from normal to a moderate iron overload, usually below levels achieved in haemochromatosis. MHF is defined by high serum levels of ferritin with normal transferrin saturation and metabolic dysfunction. In MHF, hepcidin is released in response to iron stores and the ability of the hepcidin to downregulate intestinal iron absorption is generally preserved.

## 4. Current Treatment Strategies for Iron Overload

### 4.1. Phlebotomy

The gold standard for the treatment of haemochromatosis is phlebotomy, based on the removal of red blood cells (RBCs) to decrease serum iron levels. This treatment does not clinically improve hemochromatosis, but it can prevent complications, especially in patients with organ alterations due to iron overload. Furthermore, the available data suggests that phlebotomy may improve liver function tests, and result in the regression of liver fibrosis and cirrhosis in a subset of patients. Data about the effect on the risk of diabetes or cardiovascular disease is still lacking. Hence, therapeutic phlebotomy is the preferred treatment for blood disorders. The morbidity and mortality of patients with haemochromatosis are reduced when treatment is started before cirrhosis and/or diabetes develops [38]. The Danish 2019 guideline promoted serum ferritin levels of <100 lg/L in the induction phase, and serum ferritin levels of 50–100 g/L and transferrin saturation of <60% in the maintenance phase. The American College of Gastroenterology recently defined 50–100 g/L as the target serum ferritin level for patients with haemochromatosis [39,40]. Meanwhile, an international group of experts recently published a practical recommendation for the therapeutic aspects of HFE in 2018, proposing a ferritin level of 50 lg/L as a target for the induction phase, and a range of 50–100 lg/L for the maintenance phase. Experts of the Delphi panel recommend that treatment ferritin should be in the ranges of <200 lg/L for women and <300 lg/L for men during the maintenance phase. Reducing ferritin concentrations down to 50 g/L during the maintenance phase is poorly tolerated in elderly patients. The volume and frequency of phlebotomies are usually 400–500 mL weekly or every 2 weeks during the induction phase, based on the patient’s body weight and patient tolerance, and every 1 to 4 months during the maintenance phase, according to the patient’s iron condition. This treatment is unsuitable for iron-loading anemics or for patients with severe comorbidities such as heart disease. Iron chelation therapy is recommended in these cases. Three different kinds of chelators are currently available in Europe and the United States: deferoxamine, which is administered parenterally, and the oral chelators deferiprone and deferasirox, which are characterized by longer plasma half-lives [41,42]. Iron chelation therapy has shown good results in reducing the iron load and improving survival in patients with transfusion-dependent anemia.

### 4.2. Hepcidin Agonists

Treatment with hepcidin agonists aims to prevent or alleviate iron overload in hepcidin deficiency diseases, especially in cases of hereditary hemochromatosis and β-thalassemia. Hepcidin can be increased in two ways: by stimulating endogenous hepcidin production or by administering exogenous hepcidin or hepcidin agonists. Several hepcidin mimetics have been shown to prevent iron overload, such as minihepcidins, small peptides engineered to mimic the function of hepcidin, while simultaneously inhibiting ferroportin activity. In hepcidin-deficient mouse models, minihepcidins prevent liver iron accumulation; on the other hand, when minihepcidin is administered in cases of preexisting iron overload, they promote the partial re-distribution of iron from parenchymal cells to macrophage stores. Rusfertide is a synthetic hepcidin mimetic and helps to reduce transferrin saturation and serum iron levels [43]. Rusfertide could effectively replace phlebotomy without worsening symptomatic iron deficiency [44]. Moreover, the synthetic hepcidin mimetic LJPC-401 was evaluated in a phase II randomized, placebo-controlled study; proving a significant reduction in transferrin, consequently reducing the amount of phlebotomy sessions. Other hepcidin agonists in clinical trials include the small molecule VIT-2763 [45], as well as antisense oligonucleotides and siRNAs targeting TMPRSS6/matriptase-2, which increase endogenous hepcidin production.

### 4.3. Iron and Sepsis

During sepsis, pathogens try to take iron from the host to promote their proliferation and survival, causing alterations in the host’s iron metabolism [46]. This results in an increase of iron uptake in cells, simultaneously causing increased iron-driven oxidative injury and cell death. During infection, iron may be sequestrated, which is characterized as a defense mechanism, as bacteria requires iron to grow. The harmful effects of iron during infection have been linked to elevated serum ferritin and non–transferrin-bound iron levels. Furthermore, during infection, cytokines increase and hepcidin levels quickly rise, resulting in a reduction in plasma iron concentration. Xiyang Zhang et al. showed that ferritin and hepcidin concentrations were significantly elevated in intensive care unit (ICU) patients, and their values were the highest in sepsis patients [47]. Recently, therapeutic strategies aiming to combine host-directed therapies with pathogen-directed treatments have been developed, minimizing drug toxicity while attempting to reduce antimicrobial drug resistance [48,49,50]. The blocking of iron sequestration has been proven to be a promising therapeutic strategy against bacterial infections. Recently, a new therapy allowed the access of mycobacteria in iron to be reduced, based on the inhibition of the synthesis of siderophores released by M. tuberculosis [51].

### 4.4. Assessment of Iron Status

The study of iron metabolism and homeostasis mainly relies on some historical tests: serum iron, transferrin (Tf), total iron-binding capacity (TIBC, calculated as Tf × 1389), transferrin saturation (TSAT, calculated as serum iron/total iron-binding capacity × 100), and serum ferritin [52]. These tests are traditionally used in the evaluation of iron status and the diagnosis of iron deficiency anemia (IDA). Despite the threshold of ferritin level that defines iron deficiency remaining unclear, the reference definition of iron deficiency is the absence of stainable reticular iron in a bone marrow smear. A recent review [53] comparing ferritin with absent bone marrow iron reported that a ferritin threshold value of <15 mg/L had a sensitivity of 59% and specificity of 99% for iron deficiency, while a ferritin threshold value of <45 mg/L had a sensitivity of 85% and a specificity of 92% [54]. According to the World Health Organization, iron deficiency (ID) in the general population is defined as low serum ferritin levels (<15 ng/mL) and decreased TSAT (<16%), which are used to diagnose iron deficiency (ID) and (IDA) in patients without inflammation at the same time [55] Even though the gold standard for diagnosing ID is bone marrow aspiration with specific staining of iron, it is limited by its invasive nature and cost. Meanwhile, the current American and European guidelines on heart failure define iron deficiency according to the following parameters: serum ferritin levels of <100 ng/mL or the combination of ferritin between 100 and 299 ng/mL and a transferrin saturation (TSAT) of <20%; [56] (Table 1). More recent iron metabolism biomarkers may assist in redefining the risk associated with ID. The soluble transferrin receptor (sTfR) is produced by the proteolysis of the membrane transferrin receptor (TfR). This parameter is related to the expansion of erythropoiesis or iron deficiency and its release into the bloodstream is increased during iron deficiency. Derived parameters, such as the TFR/log ferritin ratio, are evaluated to diagnose iron deficiency in inflammation, while the transferrin saturation (Tsat)/log hepcidin ratio is used to suspect iron-refractory, iron-deficiency anemia (IRIDA) [57] (Table 2). Enzyme-linked immunosorbent assay kits can measure serum hepcidin levels. However, hepcidin levels rapidly change in case of activating and inhibitory signals. For this reason, some researchers propose that the concentration of hepcidin levels should be determined, as this can influence the choice and better administration of iron supplementation [58]. Other biomarkers of iron status include the reticulocyte hemoglobin content (CHr) and the percentage of hypochromic red blood cells (%Hypo). CHr provides an expression of iron availability for erythropoiesis within 3–4 days and CHr < 27.2 pg is a diagnostic marker for iron deficiency. %Hypo determines the concentration of hemoglobin in red blood cells (RBCs), which reflects the absolute amount of hemoglobin and the RBC size and serves as a sensitive marker of iron deficiency and reflects iron changes in long-term assessments [59]. %Hypo-He, with a cutoff value of 0.6%, is a potential parameter with high sensitivity and specificity for evaluating iron status. This parameter has a faster turnaround time than biochemical markers [60]. During iron supplementation, CHr can normalize within 2–3 days, whereas %Hypo can take months to do so [61]. Ret Hb equivalent is used to assess the severity of iron deficiency in patients. With a Ret Hb cutoff level of 27.2 pg, iron deficiency could be diagnosed with a sensitivity of 93.3%, and a specificity of 83.2% [62,63].

## 5. Conclusions

Currently, iron assessment allows to classify two main clinical conditions: iron deficiency and iron overload. There are different historical tests and new biomarkers that influence this aim, informing diagnosis procedures regarding alterations in iron metabolism. The latest biomarkers, in addition to availability of serum iron, could also provide information about iron cycles in the blood andthe risks of developing anemia or hemochromatosis. Knowledge of molecular pathways, where iron is implicated, allows to expand the research field, including the study of ferroptosis or ferritinophagy, where many molecules are involved in the same way in different mutations, and this could explain their involvement in tumor genesis or other pathologies. A promising field of research is the relationship between the amount of body iron and bacterial infections, which is a current problem in the health field. This could be an auspicious source, providing clinicians with the knowledge and means against bacterial infections, especially in the most critically ill patients. Finally, alterations in iron metabolism could help in the study many metabolic pathologies related to iron metabolism, from uptake to release, allowing the development of increasingly new personalized drugs for specific molecular mutations, limiting the incidence of some pathologies that are still considered as health emergencies.

## Figures and Tables

**Table 1:** Diagnosis of iron deficiency.

Parameters	Threshold	Reference
World Health Organization’s parameters		
Serum Ferritin	<15 mg/L	[54]
TSAT	≤16%	
American and European Guidelines on Heart Failure		
Serum ferritin	<100 ng/mL or between 100 and 299 ng/mL	[55]
(TSAT)	<20%;	

Comparison of the thresholds utilized to diagnose of iron deficiency between the World Health Organization parameters and the American and European Guidelines on Heart Failure. Abbreviations: TSAT: transferrin saturation.

**Table 2:** New iron parameters for diagnosis of iron deficiency.

Parameter	Function	Threshold
Soluble transferrin receptor (sTfR)	Related to the expansion of erythropoiesis and its release into the circulatory system increases during iron deficiency.	There is no standardized or validated cutoff value for sTfR.
Transferrin/log ferritin ratio	Evaluated to diagnose iron deficiency in inflammation settings.	1.70
Transferrin saturation (Tsat)/log hepcidin ratio	Evaluated to suspect iron-refractory, iron-deficiency anemia (IRIDA).	5.6%/nM
Hepcidin	Allows one to choose the better administration of iron supplementation.	There is no standardized cutoff value.
Reticulocyte hemoglobin content (CHr)	Provides iron availability for erythropoiesis within 3–4 days.	<27.2 pg
Percentage of hypochromic red blood cells (%Hypo)	IA-sensitive marker of iron deficiency and changes in long-term assessments.	0.6%
Ret Hb equivalent	A marker for determining the presence and severity of iron deficiency in different patients.	27.2 pg

New markers used to diagnose iron deficiency, referring to their usefulness and relative cutoff values. Abbreviations: Ret, reticulocyte; Hb, hemoglobin.

## Data Availability

No new data were created or analyzed in this study. Data sharing is not applicable to this article.
